# The effects of tech and non-tech innovation on brand equity in China: The role of institutional environments

**DOI:** 10.1371/journal.pone.0215634

**Published:** 2019-05-08

**Authors:** Qiong Yao, Liwen Huang, Mingli Li

**Affiliations:** School of Management, Jinan University, Guangzhou, Guangdong Province, China; University of Toronto, Rotman School, CANADA

## Abstract

Recently, innovation has been a key driver of brand equity. However, the emerging economies provide a dynamic institutional environment that makes it difficult to explore the relationship between innovation and brand equity. By combining the brand equity literature and institutional theory, our research investigates the effects of technical and non-technical innovation on brand equity and how the effects vary within different institutional factors (product market development, regional legal environment). A sample composed of 124 listed companies in China from 2009 to 2014 was analyzed empirically and provides strong support for the theoretical predictions. The results confirm the positive effect of the two innovations on brand equity and the contingent effect of institutional factors as follows: the regional legal system positively moderates the relationship between the two innovations and brand equity, and product market development positively moderates the relation of technical innovation and brand equity; there was found to be no significant influence of non-technical innovation on brand equity. This study provides crucial theoretical and managerial implications for managers.

## 1 Introduction

In contemporary business, enhancing brand equity has been a key strategic issue for many firms attempting to offer superior returns instead of providing initiatives of potentially lower value “but with more immediate and quantifiable financial outcomes” [1–3]. More managers are inclined to forgo short-run profits for maximum long-term added value by offering brand name products or services, which is referred to as brand equity [4–6]. As a key driver of brand equity [6], innovations can create differentiation, enhance a brand’s value proposition, and revitalize the brand [2]. Furthermore, a brand’s investment in innovation may grant it the ability to successfully employ a wider range of marketing strategies than the competition [7]. Therefore, building an innovative mechanism capable of sustained growth may be necessary for firms to affect brand construction positively. However, the emerging economies are experiencing rapid changes, and this provides a paradoxical environment to the development of innovation and brands [1, 8]. Differing economic, social and legal institutions require multiple emphases on different things in different markets [1]. Compared with developed countries, the brand equity of Chinese firms is generally in low level, characterized by lack of social value expression and price premium as well as terrific unevenness across the regions [9, 10]. Thus, it is still unclear how innovation behavior fosters firm’s brand equity in emerging economies [1, 11].

According to brand equity theory, from benefit views, innovation is one of fundamental ways to promote brand equity by clarifying core benefit for the brand [6]. The innovations, such as the inclusion of significant new product or service attributes, can bring customers functional benefits, experiential benefits and symbolic benefits [6]. For example, eco-friendly innovations usually occur on the new changes of material reduction and recycling, energy-saving, emissions reducing [12], which conveys functional benefits of health, cost and energy savings, experiential benefits of positive emotions and high consumers’ satisfaction, and symbolic benefits of care for the environmental consequences and identification of ‘green consumers’.

However, in reality, given the long-term and cost of innovation [13], it’s uncertain that innovation can promote brand equity. Though the return that firm’s innovation brings is high, its performance needs a long-term innovative accumulation to clarify, because it takes time to make consumers understand and buy the innovation product or service [14–16]. In China, LeSee automobile, which focused on new changes on transportation ecosystem can’t keep the brand’s promotion lasting because of its short-term innovation since 2014. But BYD Auto, the new energy automobile manufacturer, definitely depends on long-term R&D efforts on battery technology and clean-energy ecosystem, which has resulted in many irreplaceable patents [17].

The academic literature is similarly muddled on the relationship between innovation and performance, whether the financial (profit, turnover and costs) or nonfinancial (satisfaction, perception and brand attitude) measures [18]. As to the financial performance, prior studies illustrated that the positive effect of innovation on a firm’s performance through a long-term meta-analysis [19, 20]. But some authors demonstrated that innovations have little or no impact on firm value, even negative [21–23]. For non-financial measurement, some scholars suggested that high customer satisfaction and brand reputation of an organization could be enhanced by innovation [2]. Zhang [18] also argued that the degree of innovation exerts positive influences on customer equity and brand image, but the relationship can be moderated by product category and nationality. Indeed, concerned the institutional factors in emerging economies, dysfunctional competition can directly affect innovative outcomes [8]. That is to say, even if a firm invests in R&D, the ideal financial and nonfinancial performance both cannot be guaranteed [24].

Given the conflicting findings regarding the effect of innovation on firm performance, it is necessary to discuss the contingent constraints of the external environment, especially the institutional factors. Because most papers on innovation are conducted in developed economies, which usually have well-established institutional frameworks, previous studies have largely ignored the pivotal impact of institutional forces [25, 26]. Drawing upon the institutional theory, innovation creates value by reducing transaction costs, but value redemption usually occurs alongside the fear of opportunism [27, 28], which is characterized by industry structural instability and the unenforceability of laws in emerging economies [29]. Additionally, it is difficult for firms to achieve sufficient legitimacy in the intelligence property game and access the critical resources and fair free market under the weak institutional construction [30]. Thus, the conditions under which innovative strategies will be effective in achieving brand equity has been a paucity in the research.

**Fig 1:**
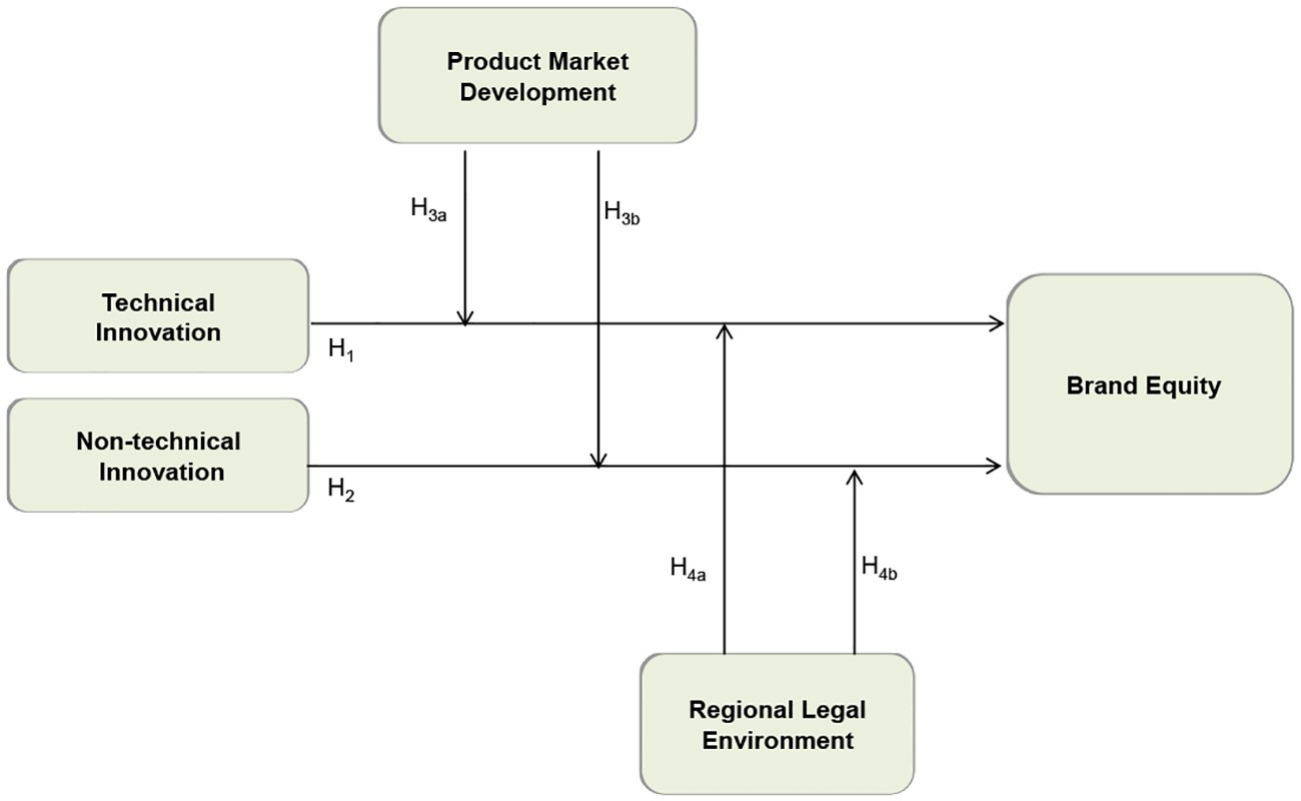
The conceptual model.

Overall, drawing upon the brand equity theory and institutional theory, this article attempts to address the two following questions: (1) Do innovation increase brand equity in emerging markets? (2) What are the institutional factors that increase or decrease the value of two-way innovation’s impact on brand equity? Specifically, we elucidate the differential effects of technical innovation and non-technical innovation on brand equity. To make these questions clear, we provide the conceptual model depicted in Fig 1 as a typical case of an emerging economy, China offers a rich setting characterized by large institutional complexity and regional heterogeneity in economic transition. Therefore, we use multiple provincial panel data from China to test our model.

## 2. Theory and hypotheses

### 2.1 Brand equity

Brand equity has been a hot issue bridging the firm and customer in *marketing* and is defined as the added value to the product by the brand name [31]. Compared with the unbranded products, firms can use their powerful brand name to attain the firm’s goals. When brand equity is high enough, it can increase the number of consumers by influencing their awareness, preference and loyalty to the firm, which results in premium pricing, lower price elasticity, repeat purchasing, trade leverage and so on [4]. Brand equity can be assessed through the three following levels of approaches: the financial level, product-market level and customer-mind level [32, 33].

First, from the financial perspective, brand equity is valued as the total sum of all cash flow (current and future) attributable to the brand, aggregating the brand’s overall franchise and licensing income [33]. Simon and Sullivan [34] calculated incremental cash flows attributable to branded versus unbranded products to measure brand equity by focusing on the stock market performance only. They are credible to senior management and the financial community, and can act as a useful guide to the value of a brand in mergers and acquisitions.

Second, from the product-market perspective, brand equity is associated with the monetary value shown in the incremental sales and profits [33]. Farquhar [31] defined brand equity as the added value attributable to the brand on tangible products, and for which the customer will pay a higher price for over the tangible asset. Hence, in extreme competition, firms with high brand equity can make use of the initiative pricing rights to occupy the market share over a generic product or its competitors [35]. In summary, in this view, the focus is on the brand-induced price premium to measure brand equity.

Finally, while some different metrics have been proposed for valuing brand equity, Leone et al. [36] indicated that “the power of a brand lies in the minds of consumers.” From the customer-based perspective, conceptualization of the brand equity is closely related to the customer’s differentiation reaction to marketing activities in line with the brand knowledge [37]. Customers can easily relate to the brand’s products, which helps customers build an emotional identity. Many studies have mentioned the specific value derived from the positive associations, awareness, loyalty, attitudes and attachments that customers have toward a brand [4–6, 37]. If a brand is rooted in the hearts and minds of the customer, the brand has the opportunity to retain and acquire customers, which suggests that customer attitudes are a precursor to customer actions. Therefore, it is indispensable that the customer mind-set measure of brand equity includes both awareness/loyalty and brand associations [32].

Compared to most of the brand equity literature evaluating which focuses merely on financial performance, our article incorporates the financial aspects with the consumer-level measurement to measure brand equity.

### 2.2 Technical and non-technical innovation

This article adopts the item of *innovation* as the implementation of an idea, whether pertaining to a device, system, process, product, or service, which is new to the organization at the time of adoption [38]. Despite some diverging arguments in the literature in terms of different economic sectors in the typology of innovation in recent decades, some consensus is reached with regard to innovation, which is considered to be a complex phenomenon that includes technical (new products and new process) and non-technical (new service and new process) aspects of innovative features [39]. Specifically drawing upon the socio-technical theory, the literature argued that it is necessary to consider the changes and new offerings by the organization in both the technical system and the social system, with the aim of optimizing the organizational performance [40, 41], and these studies categorized innovation into the two following types: *technical innovation* and *non-technical innovation* [42–44]. The former presents the major force behind structural processes, playing a pivotal role in revolutionizing the productivity, delivery and transactions methods with technology [45], which revolves around the product quality and productivity efficiency to perform innovation attached to new technical changes (product, process). However, the latter, which is defined as the enhanced offerings, focuses on the internal attention to human-interactions within the social system pertaining to *non-technical innovation* (service) [39]. Differing from technological-enabled innovation, non-technical innovation delivers goods and services primarily by humans, typically involving interpersonal contact with customers, which can be perceived as the brand having better service value than technical innovation.

The majority of the literature of innovation has been aimed at technical innovation in the manufacturing sector, while ignoring the role played by non-technical innovation in the competition strategies of firms, especially when the service sector has become increasingly important to economic development worldwide [46, 47]. In addition, innovation in the human-dominant level of a firm may not follow the technological trajectory [48]. Despite a growing number of studies recognizing the importance of non-technical innovation recently, the extant studies still know little about whether non-technical innovation complements technical innovations and has transformative abilities on firm-level outcomes [48].

According to the above arguments, in this article, we identified the extent to which technical innovation and non-technical innovation enhance the brand propositions of firms and the consumer’s perception of the brand itself, respectively. Our data in this study are cross-sectional, which represents the importance of longitudinal studies in understanding the management of innovation, especially in the manufacturing industry [49].

### 2.3 Institutional environments in China

According to North [50], institutions are “the rules of the game in a society or, more formally, are the humanly devised constraints that shape human interaction.” Institutional theory is preeminent in helping to explain the impacts on the strategies of enterprises. This is especially useful because government and societal influences are stronger in these emerging economies than in developed economies [51].

Drawing upon the efficiency-based view rooted in institutional theory, the role of institutions is to reduce transaction costs, which are the value creation generated by innovation, through reducing uncertainty and establishing a stable structure that facilitates interactions [51]. That is, the transaction cost increases because those involved in the transaction have to overcome the hazards of opportunism while facing the conditions of structural instability of the industry, unverifiable information, and unenforceable laws [28, 29]. In practice, innovation requires more financial commitment, which is risker, and accrues returns in the long term [52]. In the environmental context of emerging economies, especially China, which is in dynamic transition, characterized by the “nested institutional hierarchy” of firms and government agencies as well as other institutions [53], the weakness of the legal system and the unsustainability of the industry may not help firms economize the transaction costs, which restrains innovation.

The findings from the two dimensions of institutional theory suggest that the market orientation and legal orientation are related to managerial decisions and performance. First, legitimacy can also help firms obtain access to valuable resources to survive in the marketplace [54, 55], whereas the lack of legitimacy can render survival and development difficult, such as suffering the infringement of intellectual property [56]. Despite the acceptance of the institutional environment as a significant factor in shaping the firm’s strategic practices [57], the contingent impact of institutions on the relationship between innovation and brand equity remains largely unexplored.

Second, product market development still varies markedly across regions in China [58, 59]. After more than two decades of dramatic economic transition, even though China is changing from a centrally planned to a market-driven economy, the domestic market integration remains incomplete [59]. Due to the “invisible hand,” differences in subnational regional policies not only impact the unevenly developed infrastructure but also account for resource allocation in regional economic growth [48, 58]. Thus, regions with underdeveloped economic institutions may pose challenges regarding enacting the firm’s strategies [60]. Thus, in this study, we explored the role of product market development in the relationship between innovation practices and brand equity.

As a result of the changing institutional factors, how the relationship of firm’s innovation and brand equity changes in China’s paradoxical context becomes more imperative to brand researchers.

### 2.4 Hypotheses

#### 2.4.1 The effect of technical innovation on brand equity

According to Damanpour [61], technical innovations occur as a result of the use of a new tool, technical device, or system, with the new elements introduced into the production system generating product improvement. As mentioned in the prior research, technical aspects of innovation consist of product innovation and process innovation [62–64], and technical-based service innovation named e-innovation [46], such as remote, intelligent and separate services through the use of Internet technology [65, 66]. More generally, technical innovation contributes principally to the productivity efficiency and product quality for long-term competitive advantages, which are embedded in brand equity. Specifically, regarding the two perspectives of brand equity, we demonstrate the relationship of technical innovation and brand equity as follows.

One is the firm-level view, generally found to emphasize the marketing and financial outcomes affiliated with the brand. Blundell [67] suggests that new product introductions have a positive impact on the market value and profitability of a firm. Regarding technical process-innovation, productivity and product quality both gain, thus it allows firms to be able to respond to competitive products from the cost side, i.e., production, process, and transaction [45]. Moreover, the firm may benefit from a price premium, which shows that customers are willing to pay more for the product or brand [2, 68]. Then, a third practical result is that the firm can more easily create a new market if it successfully conducts the implementation of an idea as a new product, thereupon the market share and sales increase [69]. As a result of technical innovations over time, firms can increase market share and financial revenue, followed by revitalization of their brand [70].

Customer-based brand equity (CBBE) is defined as the added value consumers derive from the brand name, and it may come primarily from the positive associations, awareness, loyalty, and perceived quality of the brand [4]. Scholars found that technical innovations, such as the inclusion of significant new product attributes, can increase the consumer evaluation of quality by improving the favorability and strength of associations and clarifying the core benefits for the brand [6]. The adoption of technical process and product improvements makes it possible to satisfy the consumers’ changeable needs immediately, as well as reducing the customer’s cost of information processing and trade-off, nevertheless competitors not [45]. Additionally, consumers may recognize convenience and comfort from the new technological attributes and will probably appreciated the innovation efforts and abilities, thus creating a better image for the brand [18]. Satisfying the customer’s needs instantly and product quality are the pivotal determinants that impact the consumer’s purchase decision and experience, thus improving the evaluation of the brand (in forms of price, sale and revenue premium) over time [2].

Overall, there is an integrated view of brand equity (firm-level and consumer-level views) that states that technical innovation has a stronger impact on how the consumer perceives the brand proposition, then contributes to consumers willing to pay more, which means that the firm has price power (price premium strategy) in the market. The two aspects of financial performance and consumer perception may promote the brand equity through technical innovation. Thus, we predict the following:

**H**_**1**_: Technical innovation relates positively to brand equity.

#### 2.4.2 The effect of non-technical innovation on brand equity

Recently, studies have reviewed the non-technical traits attached to human-dominant services and have increasingly focused on the interaction between firms and customers (dialogue and participation) [47]. The differences in traits between technical and non-technical innovation suggest differences in the determinants. Technical innovation is usually related directly to firm-level output because of cost and scalability advantages, but potential technological limitations or bugs may lower customer-level outcomes, such as satisfaction/loyalty in the short run [46]. Conversely, non-technical innovation may have a greater influence on customer-level elements such as awareness, evaluation and satisfaction of the brand [46]. Additionally, a number of studies found that non-technical innovation can have a strong impact on financial performance [71–74].

First, the human-dominant nature of non-technical innovation makes it a valuable asset for generating sustainable competitive advantages to satisfy the consumer mindset. In the frame of non-technical innovation, tight interpersonal contact in product and service creates tight company-customer relations. In particular, the repeated human interactions in human-dominant industries, such as hotels, catering and airline services, can enhance the short-term customer experience because people-enabled innovation can resolve customer problems in a timely manner, thus helping firms recover from service failure [46]. Some scholars have stated that non-technical innovation creates collaborative value with joint innovative alliances that positively impact customer care, and this newly introduced interpersonal contact helps the consumer orientation to hold in the long-term relationships with clients [31, 75]. Thus, we propose that the link between the innovative endeavor and motivation is recognized by customers.

Additionally, it is possible for firms to earn a reputation due to strengthening interpersonal abilities, which is difficult for competitors to replicate. Along with the innovation application, it allows customers to become aware of organizational learning capabilities and the knowledge-sharing value of firms, and this collaborative mechanism can help the firm to shape their structure and with client relationship management [76]. In addition, non-technical innovation may exhibit features such as inseparability and heterogeneity. Inseparability is due to simultaneous production and consumption of the service [77], and heterogeneity of the new service or product offering is due to inconsistency in human performance, which helps build the unique value delivery system that can enhance the customer perception, and it finally improves the consumer’s beliefs about brand. Therefore, we predict the following:

**H**_**2**_: Non-technical innovation relates positively to brand equity.

#### 2.4.3 The moderating effect of product market development

*Product market development* grasps the degree to which market transactions are formed by free market mechanisms and trade barriers constrained by local market protectionism, and it represents the level of market-oriented economic mechanism across different regions. The Chinese economy has undergone a dramatic transition from center planned to market orientation along with industrial turbulence and a cross-sectional governance dilemma. Because of the “iron hand” on crucial industries, territorial industrial structures and product market development vary tremendously across the country, which results in trade protectionism enforced by the local government and institutions. It then begets market fragmentation and jeopardizes the development of free market mechanisms. As a result, there is a greater deal of diversity in the product market across regions in China than among developed countries. In this study, we posit that in a highly developed product market, the relationship between innovation and firm performance can be stronger, and our discussion will be organized in the following aspects.

Initially, when product market development is static, trade protectionism reigns over the market imposed by local government, and then transaction costs increase, including search, contracting, monitoring and enforcement costs [28, 50, 78, 79]. Due to the hazards of market opportunism, the market-supporting institutions directly affect the firms can access critical market resources to make innovative activities [30]. Obviously in China, which has an incomplete market-supporting system, the high cost of transactions does not enable firms to access and leverage critical resources such as information, financial assets or human talent. Specifically, it does not allow firms to absorb technologies and technological capabilities, thus the promotion of productivity and product quality is minimal, due to the high costs of developing and testing the new technological products [80]. Similarly, when firms expect to attain non-technical innovative performance, the underdeveloped product market usually hinders its knowledge-sharing mechanism and organizational learning ability in building their brand image [43].

Second, a product market orientation is posited to lead to greater customer satisfaction [81]. In a static product market, there are many unfilled gaps in the marketplace, so consumers care more about the product availability and affordability and less about product variety and attractiveness [81]. As a result, firms focus more on supply sufficient products and services rather than to satisfy new market demand and consumer preference by innovative activities. In addition, at this stage the firms may have not many competitors in the marketplace due to institutions’ intervention. Jaworski and Kohli [81] mentioned that in the case of low competition, an organization cannot provide many alternatives for customers, as well the consumers pay less attention to the new benefits, therefore the organization is likely to lose them.

In contrast, with the more development, the weakened trade protectionism can provide opportunities for firms to access multiple resources and knowledge, whatever the novelty of technologies, technological abilities or newly generated organizational and marketing methods. It helps firms to access more critical resource and apply on innovation process [82]. Therefore, the transaction cost decreases due to the relatively fair market-support institutions, and the firm’s innovation decision enables them to gain a price premium.

In this period, market demand and customer preference become more dynamic. How to satisfy increasingly changing customer preferences and gain the first mover advantages from competitors has been a focus for companies.

What’s more, the saturated market instead intensifies competition, firms must build dynamic core competences that can be defended and strengthened [30]. Organizations in highly competitive environments face more pressure, and then focus more on learning about competitors [83]. Thus innovation becomes growing and innovative outcomes can create opportunities for brands to grow and expand into new areas, which could help the firm circumvent hypercompetition. In response to fierce rivalry, technical or non-technical innovation becomes essential for the firms’ survival, success, and renewal [84]. Particularly, the technical innovation may lead to many new products and processes changes that advance the industry’s efficiency [82]. This efficiency of evolution has mirrored the changes seen in non-technical innovation, which can help to reinforce professional and customer-friendly images of the marketing design and delivery. In less-controlled product markets, a firm’s technical innovation or non-technical innovation is likely to strengthen firm-level and customer-level brand performance and earn an aggressive reputation in the marketplace. Therefore, the following can be posited:

**H**_**3a**_: Product market development positvely moderates the effect of technical innovation on brand equity.
**H**_**3b**_: Product market development positvely moderates the effect of non-technical innovation on brand equity.

#### 2.4.4 The moderating effect of the regional legal environment

The regional legal environment regulates the production, exchange and distribution in a sample of basic political, social and legal basis rules across the regions [85]. No firm-level strategical behavior is immune from the legal institutional framework in which it is embedded because they are also a reflection of the formal constraints of the particular institutional system that decision makers face [50, 83]. Prior researchers have suggested that the legal environment is a key determinant of innovations and the performance of these innovations [5, 86, 87]. What we observe under China’s imperfect governing is that the lack of patent protection and legitimacy will put the firms at great risk and cost, and the legal constructions are asymmetrical across regions.

As mentioned above, innovation is fraught with risk and uncertainty, which raises the transaction cost. However, the function of law and regulation is to reduce them. With weak legal protection, a victim of opportunistic conduct has very little legal recourse for protecting their innovations [29], thus privileged locals may follow a “hit and run” imitation strategy to steal innovative outcomes, which naturally stimulates the firms’ desire for a “patent fence” because it can preempt R&D rivals by hindering their ability to introduce substitutes and compete with its “core” innovations [88]. Hence, innovation under unenforceable law is especially sensitive to copyright infringement. Absence of the exclusive right leads to failure in value creation, which evolved in the commercialization of technical and non-technical innovation. To survive the exploitation of intellectual property, firms generally pay high transaction costs. Firms encounter not merely marketing threats full of adulterated substitutes, but the dilemma of confusing the consumer with new products and services. Researchers stated that firms which adopt the “hit and run” strategy may build rapid replication abilities based on imitation, so the threats will increase if innovators are unable to establish effective brand cognition in weak legal environment [89].

In addition, firms cannot acquire legitimacy in strong legal-support institution. Generally, firms which adopted innovative behaviors expected to be recognized by customers and stakeholders [90]. In fact, pure novel actions and ideas cannot register because there is no mature legal logic to interpret their appearance, thus these innovations may not be recognized and accepted by consumers [91]. Because the judgements depend on the subjective cognition of innovation-institution consistency characterized by meanings and values in the existing legal environment, rather than objective novelty in itself [91]. Then, it is possible for customers to be loyal to the incumbent brand [92]. Improvement of technical and non-technical innovative legitimacy becomes necessary in case of innovation-institution inconsistency, and a strong regional legal environment nurses the legitimacy for firms. Therefore, in the low level of a regional legal system, innovation may decrease the brand equity with the erosion of the financial benefit, and the customer’s perception and appreciation. Conversely, the perfectly constructed legal system will endow legitimacy, commercial protection, and fiscal treatment to help strengthen the brand equity. Thus, we hypothesize that:

**H**_**4a**_: The regional legal environment positively moderates the effect of technical innovation on brand equity.
**H**_**4b**_: The regional legal environment positively moderates the effect of non-technical innovation on brand equity.

## 3. Method

### 3.1 Sample and data sources

This study selected a sample of 124 listed firms from 2009 to 2014 in China. Because listed firms can perform more R&D due to solid resources and experiences and their data are more accessible and credible, we acquire the listed firms’ data. We eliminated listed firms for which the necessary financial data were unavailable; finally, we obtained 124 firms in 25 provinces with 738 observations. The cross-industrial data covered manufacturing, financial, real estate, software and information and technology industries, etc., in line with the National Industries Classification (GB/T 4754–2011) conducted by the National Bureau of Statistics of China.

To carry out the empirical analysis, we use a unique cross-sectional panel dataset from multiple sources for minimizing the common method bias of crucial variables [46], including primary data coded from the corporate annual reports and social responsibility reports and secondary data from diversified channels. Table 1 shows the variables, measures, and data sources in this paper.

**Table 1:** Variables, measures, and data sources.

Variable	Abbreviation	Operational Measure	Data Source
*Focal Variables*			
Technical innovation	TI	Annual firm-level count of technological innovations	Annual reports; Social responsible reports
Non-technical innovation	NI	Annual firm-level count of service innovations	Annual reports; Social responsible reports
Brand equity	BE	The index of brand equity	World brand laboratory
Product market development	PMD	The third sub-index—regional legal environment of Marketization Index of China’s Province	Marketization Index of China’s Province (NERI data set)
Regional legal environment	RLE	The fifth sub-index—regional legal environment of Marketization Index of China’s Province	Marketization Index of China’s Province (NERI data set)
*Control Variables*			
Firm age	FA	Number of years the firm had been in operation	CSMAR
Firm size	FS	Number of firm’s total employees	CSMAR
Firm growth	FG	The rate of increase of main business revenue	CSMAR
Ratio of independent directors	INDR	$\frac{The\;number\;of\;independent\;directors}{The\;number\;of\;board\;of\;directors}$	CSMAR
Regional investment in the fixed assets	RIF	The index indicates that the gross investment in the fixed assets of provinces	National Bureau of Statistics CSMAR
The price index of regional investment in the fixed assets	PIRIF	The index indicates that the fluctuation of invested commodity prices	National Bureau of Statistics CSMAR

First, we focus our investigation of technical and non-technical innovation on publicly disclosed listed firms with annual reports and social responsibility reports (CSR) over a 6-year period, from 2009 to 2014. Then, we obtain the data of brand equity from World Brand Laboratory’s annual survey, which is the leading representative consultancy of worldwide brand evaluation. World Brand Laboratory was set up in New York City in 2003, and it offered branding advising for top corporations, organizations, academic institutions and governments, such as China Mobile, State Grid, Air China, China Life, CCTV, People’s Daily, Haier and Lenovo, etc. It issued ‘*The China’s most influential brands*’ per year. Compared to most brand-evaluating institutions who focus only on the financial assets of the brand, World Brand Laboratory includes the brands’ intangible assets appraisal, integrated with financial status, customer behaviors, and brand strength.

Finally, we obtain the data from NERI dataset, CSMAR as well as Sina financial dataset. The Marketization Index, constructed by NERI, which ranks the Chinese provinces according to their level of marketization. This comprehensive measure reveals each province’s economic, financial, political, and legal development. The NERI Marketization Index contains five primary categories: (1) government and market relation, (2) non-state (private) economy development, (3) product market development, (4) factor market development, and (5) the regional legal environment. Each of the five components (including these two key moderators) contains several sub-indices based on primary survey data collected by NERI and archival data from the National Bureau of Statistics of China. Prior studies have used this index widely for academic research and policy analyses [93, 94].

### 3.2 Measures

#### 3.2.1. Innovation

Consistent with prior studies [46], we used content analyses to collect data on technical and non-technical innovation of the sample firms. Content analysis is a research technique for the objective, systematic, and quantitative description of the content of communication. We adopt this methodology in this paper, which is derived from journalism, political science and social psychology [95].

This method was chosen to measure innovation for two reasons. First, traditional measurements of innovation, such as R&D input, output or patents, may not include some non-technical innovations, such as a newly introduced airline or a new human-enabled service. Second, not all corporations would disclose their R&D information, leading to data gap in annual reports and social responsibility reports. To resolve the data gap and absence of non-technical innovation information, as a method of studying and analyzing communication in a systematic, objective, and quantitative manner for the purpose of measuring variables [96], content analysis is usually used in news communication, then spreads to marketing research [97, 98].

We captured sample data from publicly listed firms’ annual reports and social responsibility reports in 2009–2014, which may obtain legitimacy among outside constituents [99]. In line with the definition, the contents were classified with two categories: 1) the new product or production process with the technological change [23]; 2) an intangible new or improved offering by personnel interaction [46]. Through these valid key terms such as “new”, “innovation”, “technology”, “process” and “service”, categories related to technical innovation and non-technical innovation emerged and then we coded them.

To remove the bias of chance, this study adopted the method of Scott’s pi (π) and Cohen’s kappa (k) to estimate the two inter-coders reliability of content analysis [100]. First, Scott’s pi (π) is the ratio of the actual difference between obtained and chance agreement to the maximum [101]. It can be roughly interpreted as the extent to which the coding reliability exceeds chance. $Scott^{\prime }s\;pi(\pi )=\frac{P_{0}-P_{e}}{1-P_{e}}$. Where P_0_ (observed percent agreement) represents the percentage of judgments on which the two analysts agree when coding the same data independently; and P_e_ is the percent agreement to be expected on the basis of chance. In this paper, P_0_ is 0.98, P_e_ is 0.67, and pi is 0.94, which shows that the reliability is acceptable (p<0.01).

Second, a coefficient recently proposed by Cohen [102] is also quite similar to *pi*, which is optimized on the basis of Scott’s pi. The equation is as follows: ${Cohen}^{\prime }s\;kappa(k)=\frac{P_{0}-{PA}_{e}}{1-{PA}_{e}}$. Where *P*_0_ also refers to the percent agreement and is different from *P*_*e*_, *PA*_*e*_ shows the difference in the distribution of the two coders by multiplication rather than addition. Therefore, k is simply the proportion of chance-expected disagreements which do not occur, or alternatively, it is the proportion of agreement after chance agreement is removed from consideration. In this paper, *P*_0_ is 0.98, *PA*_*e*_ is 0.667, and *pi* is 0.944, which shows that the reliability is acceptable (p<0.01). The results both show that the boundary between the technical innovation and non-technical innovation is clear and precise. In total, we obtained a valid sample of 11899 innovations, including 10285 technical innovations and 1614 non-technical innovations. Examples of these two innovations are shown in Table 2.

**Table 2:** Examples of technical innovation and non-technical innovation.

Firm	Year	Type	Innovation
**Haier**	2010	Technical innovation	In terms of product, launch of fluorine-free series inverter air conditioning, iot series air conditioner, leading the market demand
**Haier**	2010	Non-technical innovation	To meet user demand and improve customer satisfaction, the Haier water heater launched "full worry-free" service innovation; the service life of the product does not charge a service fee.
**Sany Heavy Industry**	2011	Technical innovation	On May 18, "the world’s first crane" SCC86000TM crawler crane was introduced (3600 tons) successfully, which marked the China’s first crawler crane.
**Sany Heavy Industry**	2011	Non-technical innovation	“In February 2011, Sany Heavy Industry launched the ‘211’ service value commitment and the ‘311’ brand value commitment, pioneering the hoisting industry service brand construction”
**Air China**	2012	Technical innovation	“Upgrade the SMS platform, online ‘abnormal flight information automatic notification system within 72 hours’.”
**Air China**	2012	Non-technical innovation	“During the flight, yoga is introduced to ease the tiredness of the passengers. A trained attendant and a video demonstration are combined, making the atmosphere more professional and appealing for passengers”

From 2009 to 2012, we find that the group of technical innovation consist of 738 observations, where the lowest observation is 0 and the highest level is 365 items, and the mean is 36.69. During the same period, the mean of the non-technical innovation group is 2.19, where the highest level is 131 and the lowest level is 0.

#### 3.2.2. Brand equity

In this research, the measurement of brand equity is adopted by the World Brand Laboratory annual report from 2012 to 2014, which is a comprehensive index consists of 1) financial status, 2) customer behaviors and 3) brand strength. The equation to compute brand equity is as follows:

V=E×BI×S

where V refers to the brand equity, E is the operating earnings, BI represents the brand’s contribution to earnings, and S refers to factors which decide is a brand is strong.

Step 1: Comprehensive Analysis of sales, profit, etc. of financial data (EVA). After the analysis of sales revenue, profits and other financial data and market competition, they used the economic value added (EVA) to determine the enterprise profitability.
Step 2: Calculate the brand’s contribution to operating income with the BVA tools (brand value added).
Step 3: Investigate the brand strength based on the survey and assignment of factors, including the industry (0–20), external support (0–10), brand perception (0–15), brand loyalty (0–15), leading status (0–10), brand management (0–10), expanding ability (0–10), brand age (0–10). This qualitative analysis made from macro-environment and micro environment to reflect the future income of the brand.
Step 4: Calculate the brand equity by the following equation: V = E×BI×S.

As we observed, the mean is 186.969 billion within 738 observations from 2009–2014, and the highest value is 2563.19 billion and the lowest is 2.76 billion.

#### 3.2.3. Product market development

From the *Marketization Index of China’s Provinces* of NERI report 2016, we used the third sub-index—the product market index—to capture this variable across different provinces. This index states that two aspects can capture product market development: 1) the extent to which product prices are determined by the pure market, including the proportion of retail products, capital goods and agricultural products prices in the market and 2) trade barriers constrained by local market protectionism and to measure it, NERI adopts a set of sampling surveys to attain the ratio of trade barrier cases in local GDP when selling products in the market. The index integrated with the two indicators is high, which means the product market is well developed and the price competition is more intensified. The index of product market development varies substantially, which is consistent with various regulatory pressures by local institutions. The lowest level is 4.78 and the highest level is 9.79.

#### 3.2.4. Regional legal environment

The regional legal environment item is also obtained from the 2016 NERI reports, which is the fifth sub-index. The index is evaluated from three aspects: 1) the scale of regional market intermediary institutions, which means the proportion of the lawyers and certified public accountants (CPA) in the local population; 2) the degree to protection of producers’ legal rights, which helps guarantee the market is normally operated; 3) the degree of protection of intellectual property, which is the ratio of patent applications to the number of scientific researchers; 4) the degree to protection on consumers’ legal rights, which is measured by the proportion of consumers’ complaints on local GDP (contrary indicator).

As we observed, the level of the regional legal environment is unstable, where the lowest is 0.63, and the highest is 16.19 (standard deviation is 4.11), reflecting the uneven institutional legal construction in China.

#### 3.2.5 Control variables

We controlled for several variables that may impact the dependent variables. First, older firms may have more experiences and resources to build up brand equity, thus we included the variable *firm size (FS)*, which refers to the number of employees for potential economies of scale in R&D efforts [24]; second, we controlled the *firm age (FA)*, which refers to the years a firm has been in operation, to eliminate the lack of experience and resources of new firms. Third, we measured *firm growth (FG)* as the rate of increase of main business revenue, and finally we included *ratio of independent directors (INDR)* because of potential impacts of various structure on firm performance. Concerning the institutional factors in an emerging economy, we included *regional investment in the fixed assets (RIF)* and *the price index of regional investment in the fixed assets (PIRIF)* in the control variables list. We obtained these variables from the China Stock Market & Accounting Research Database (CSMAR) and Sina Financial database, which are open-access databases online of financial data in China.

A total of 738 observations across 124 listed firms from 2009 to 2014 appear in our model. The average brand equity in our sample increases from 2.76 to 2563.19, which demonstrates the prevalence of brand strategies for the firms in this sample during this period. Table 3 summarizes the descriptive statistics and correlations for all measures, pooled across firms and time. It shows that all the correlation coefficients of the matrix are lower than 0.688, reflecting an acceptable level of multicollinearity [103].

**Table 3:** Descriptive statistics and correlations.

	Variable	1	2	3	4	5	6	7	8	9	10	11
1	BE	1.000										
2	TI	0.078*	1.000									
3	NI	.402**	-.009	1.000								
4	PMD	-.257**	-.062†	-.251**	1.000							
5	RLE	.178**	.033	.154**	-.269**	1.000						
6	FS	.661**	.087*	.327**	-.319**	.180**	1.000					
7	FA	.036	-.007	.083*	.176**	.080*	-.068†	1.000				
8	FG	-.063†	-.057	.001	-.007	-.015	-.020	-.052	1.000			
9	RIF	-.091*	-.032	-.146**	.612**	-.009	-.175**	.201**	-.109**	1.000		
10	PIRIF	-.032	-.026	-.018	.043	-.101**	-.027	.011	.133**	-.000	1.000	
11	INDR	.094*	.137**	.002	.070+	.033	.085*	-.153**	.010	-.142**	-.008	1.000
	Mean	186.969	13.936	2.187	7.982	7.669	30743.45	16.016	.165	14034.43	101.457	2.956
	Std. Deviation	299.811	39.687	10.375	1.254	4.106	78778.25	4.617	.452	9176.651	2.956	.689
	Minimum	2.76	0	0	4.78	.63	21	1	-.703	988.315	96	.091
	Maximum	2563.19	365	131	9.79	16.19	552810	31	6.817	42495.55	108.36	.625

Note:

^†^ p < .10,

* p < .05,

** p < .01.

"Observation" refers to the combination of firm and year for which data are available. Variables with 738 observations (124 firms) appear only in the determinants equations.

## 4. Analyses and results

### 4.1 Estimation method

According to our theoretic model, the direct effect and moderating effect of innovation on brand equity is going to be investigated. To examine the moderating effect of innovation on brand equity through institutional factors, we introduced interactive terms that multiply tec/non-tec innovation with product market development, as well as tec/non-tec innovation with regional legal environment in the regression model. Given the remarkable differences in the multiple data sources, to make our model more static, the natural logarithmic form of variables is incorporated into this model except FG and INDR (measured as the ratio number), and it also enables to eliminate multicollinearity and heteroscedasticity. The equation is as follows:

$${lnBE}_{it}=\beta_{0}+\sum_{j=1}^{6}\beta_{j}{Control\;Variables}_{it}+\beta_{7}{lnTI}_{it}+\beta_{8}{lnNI}_{it}+\beta_{9}{lnPMD}_{it}+\beta_{10}{lnRLE}_{it}+\beta_{11}{TI\times PMD}_{it}+\beta_{12}{NI\times PMD}_{it}+\beta_{13}{TI\times RLE}_{it}+\beta_{14}{NI\times RLE}_{it}+\varepsilon_{it}$$

where: *i* and *t* index firm and year, respectively. *β* refers to the regression coefficients, BE is the brand equity, TI and NI stand for technical innovation and non-technical innovation, respectively. PMD is the product market development, RLE refers to the regional legal environment. Control Variables are firm age (FA), firm age (FA), firm growth (FG), ratio of independent directors (INDR), regional investment in the fixed assets (RIF) and regional investment in the fixed assets (PIRIF). ε is an error term.

Since this static model never takes into consideration dynamic hysteresis effect, the traditional ordinary least squares (OLS) method may yield biased estimates [104], thus the dynamic panel model was introduced in this paper. Considering the potential bilateral causality between the technology innovation, non-technology innovation and brand equity, there might be some endogenous problems, so that we employed the instrumental variables (IV) approach to deal with it. However in fact, the appropriate IV is difficult to find, thus we used the Generalized Method of Moments (GMM) proposed by Arellano and Bover (1995) to estimate this equation. GMM is a specific form of IV method that uses the predetermined variables and/or lag terms of independent variables as IV, which can verify the validity of IV at the same time.

There are some advantages for utilizing the GMM method. For the first, it can help solve the issues of fixed effects and endogeneity of regressors [105, 106]; Second, GMM estimator is more efficient to deal with the heteroskedasticity by using the IV estimator [107]; Third, this method can effectively improve the consistency and validity of estimation results based on the robustness check. The GMM has two types—the difference-GMM and the system-GMM, and they can be both considered in there one-step and two-step versions. Since the system-GMM is more efficient and allowing for more instruments through an additional assumption than difference-GMM, thus system-GMM was applied in our study. After some tests, we can obtain consistent estimates of the parameters of interest in which lagged values of lnBE(t-1), TI(t-2) and NI(t-2) will be valid instrumental variables in the two-step system GMM. So that, we introduced the three variables into our model, and the equation can be rewritten as following:

$$\begin{array}{l} {\text{lnBE}}_{it}=\alpha_{0}lnBE_{i\left(t-1\right)}+\alpha_{1}TI_{i\left(t-2\right)}+\alpha_{3}NI_{i\left(t-2\right)}+\beta_{0}+\sum_{j=1}^{6}\beta_{j}{\text{Control}\;\text{Variables}}_{it}+\beta_{7}{\text{lnTI}}_{it}+\beta_{8}{\text{lnNI}}_{i\text{t}}+\beta_{9}{\text{lnPMD}}_{it}+\beta_{10}{\text{lnRLE}}_{i\text{t}}+\beta_{11}\text{TI}\times {\text{PMD}}_{i\text{t}}+\beta_{12}\text{NI}\times {\text{PMD}}_{i\text{t}}+\beta_{13}\text{TI}\times {\text{RLE}}_{it}+\beta_{14}\text{NI}\times {\text{RLE}}_{it}+\eta_{i}+\varphi_{t}+\varepsilon_{it} \end{array}$$

where: *i* and *t* index firm and year, respectively. *α* and *β* refers to the regression coefficients, BE is the brand equity, TI and NI stands for technical innovation and non-technical innovation, respectively. PMD is product market development, RLE refers to the regional legal environment. *η* and *φ* refer to province-specific effect and time-specific effects, respectively. ε denotes an error term.

This hierarchical structure includes three models. Model 1 includes all the control variables: firm size, firm age, firm growth, ratio of independent directors, regional investment in the fixed assets and the price index of regional investment in the fixed assets in the regression. Model 2 adds the dependent variables, independent variables and moderators, while model 3 includes the interaction terms between the innovation and institutional factors.

### 4.2 Results and analysis

Table 4 reports the two-step SYS-GMM results of our dynamic panel data models 1 to 3. As we see the three instrumental variables, the estimated coefficients of them are significant except TI _i(t-2)_ in model 1, suggesting they are effective as the single IV. AR(2) test for second-order serial correlation are insignificant (p>0.1), therefore the error term of these models does not have the self-correlation problems. What’s more, the Sargan Test are all insignificant for model 2 and model 3 (*p*>0.1, except the model 1), is largely enable to reject the null hypothesis that excessive identification restriction is valid, thus the chosen IVs are generally effective.

**Table 4:** Two-step system GMM panel estimation regression.

		Model 1		Model 2		Model 3	
		*Coef*.	*z-value*	*Coef*.	*z-value*	*Coef*.	*z-value*
*Instrumental Variables*							
BE _i(t-1)_		.886**	48.64	.871**	32.48	.656**	6.47
TI _i(t-2)_		-.000	-0.70	-.000†	-1.92	.007*	1.96
NI _i(t-2)_		-.001**	-6.74	-.002**	-5.69	-.011**	-2.84
*Control Variables*							
lnFS _it_		.067**	4.39	.256	.67	.366**	2.39
lnFA _it_		.265**	14.88	-.059	-1.13	.139†	1.75
FG _it_		-.043**	-9.81	.012	.42	-.265*	-2.24
IDR _it_		-.293**	-3.37	.213	.89	.654	1.26
lnRIT _it_		-.089**	-3.63	-.086	-.98	.304	1.24
lnRIP _it_		.378*	2.52	-1.837*	-2.21	-.507	-0.54
*Focal Variables*							
lnTI _it_	H_1_			.034**	2.76	.034*	2.36
lnNI _it_	H_2_			.064**	2.71	.080*	2.27
lnPMD _it_				-.143**	-2.97	-.634	-1.46
lnRLE _it_				.011**	3.09	1.507*	2.21
*Moderating Effects*							
TI × PMD _it_	H_3a_					.008*	2.36
NI × PMD _it_	H_3b_					-.001	-1.06
TI × RLE _it_	H_4a_					.002*	2.13
NI × RLE _it_	H_4b_					.002**	2.76
Constant		-1.942**		-10.383*		-2.073	
Sargan Test(p-value)		0.052		1.000		1.000	
AR(1)		0.042		0.051		0.050	
AR(2)		0.516		0.319		0.776	

Note:

^†^ p < .10,

* p < .05,

** p < .01.

“TI × PMD” denotes the interaction term of technical innovation and product market development. “NI× PMD” is the interaction item of non-technical innovation and product market development. “TI × RLE” refers to the interaction term of technical innovation and regional legal environment. “NI × RLE” stands for the interaction item of non-technical innovation and regional legal environment. Sargan Test refers to the over-identification test for the restrictions in GMM estimation. The AR(2) test stands for the Arellanon-Bond test for the existence of the second-order autocorrelation in first differences

#### 4.2.1 Effects of innovation on brand equity

As reported in Table 4, Model 1 provides the relation between control variables and dependent variable, and we can observe that lnFS (*β* = .067, *p* < .01), lnFA (*β* = .265, *p* < .01), FG (*β* = -.43, *p* < .01) and IDR (*β* = -.293, *p* < .01) are significantly related to lnBE (Model 1), which shows that the volume, growth and structure of corporation can strongly affect brand associations. Regional economic development also impacts the brand images in China through regional investment in the fixed assets (*β* = -.089, *p* < .01) and the price index of regional investment in the fixed assets (*β* = .378, *p* < .01), shown in model 1.

In support of H_1_ and H_2_, Model 2 presents both the logarithms of technical innovation (*β* = .034, *p* < .01) and the logarithms of non-technical innovation (*β* = .064, *p* < .01) are in the significant determinants. Regarding the institutional factors, the regional legal environment has a positive effect on brand equity (*β* = .011, *p* < .01), whereas the product market development is negatively related to brand equity (*β* = -.143 *p* < .01). We conjecture that when the level of product market development increases, the marketing competition intensifies and the consumer’s cognition of a single brand is confused, which may increase the promotion cost of the brand in case of a loss in consumer loyalty.

#### 4.2.2 Moderating effects

With the prediction that the product market development would positively moderate the relationship between technical innovation, non-technical innovation and brand equity, we tested H_3a_ and H_3b_ in Model 3. Whereas technical innovation in product market development has a significant, positive effect on brand equity (*β* = .008, *p* < .05), non-technical innovation does not have a significant effect (*p* > .10). Thus, H_3a_ is supported, but H_3b_ is not. The technical innovation has a stronger effect on brand equity when product market development is high than when it is low. The unexpected result for H_3b_ has some plausible explanations. When the product market development is low, customers cared more about product availability and affordability, and less about product or service attractiveness, thus the non-technical innovation cannot be easily to promote customer perception and profit. Contrarily when product market development is high, the competition intensifies due to the decrease of intervention by local government. It’s difficult to prevent the imitation of those non-technical human-dominant innovation, if so, people cannot easily recognize those real core benefits across brands in the short term, then salience of competitive advantages in the brand would be lessened, while competitors stand out of the market.

**Fig 2:**
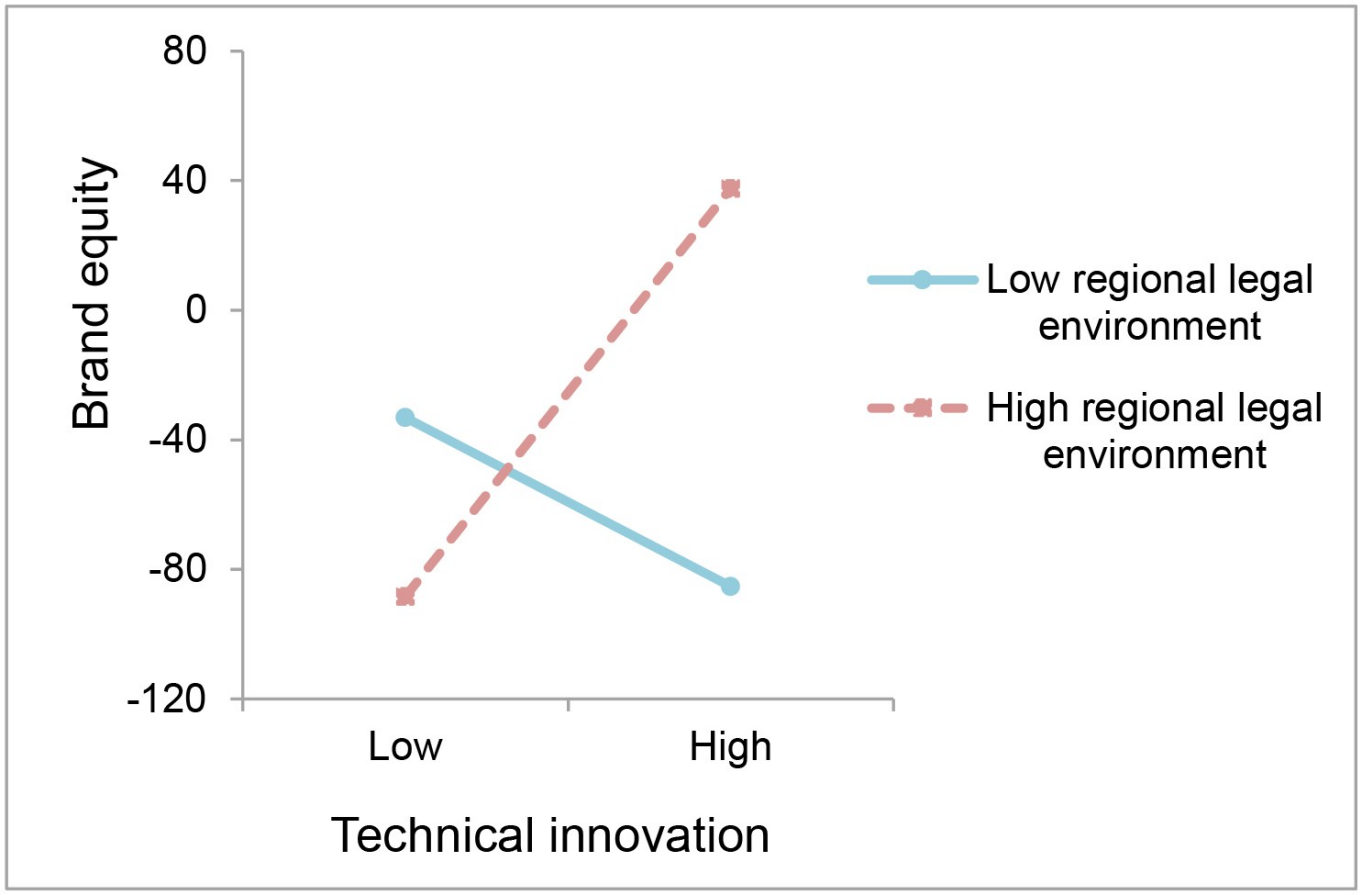
The moderating effects of H_4a_.

**Fig 3:**
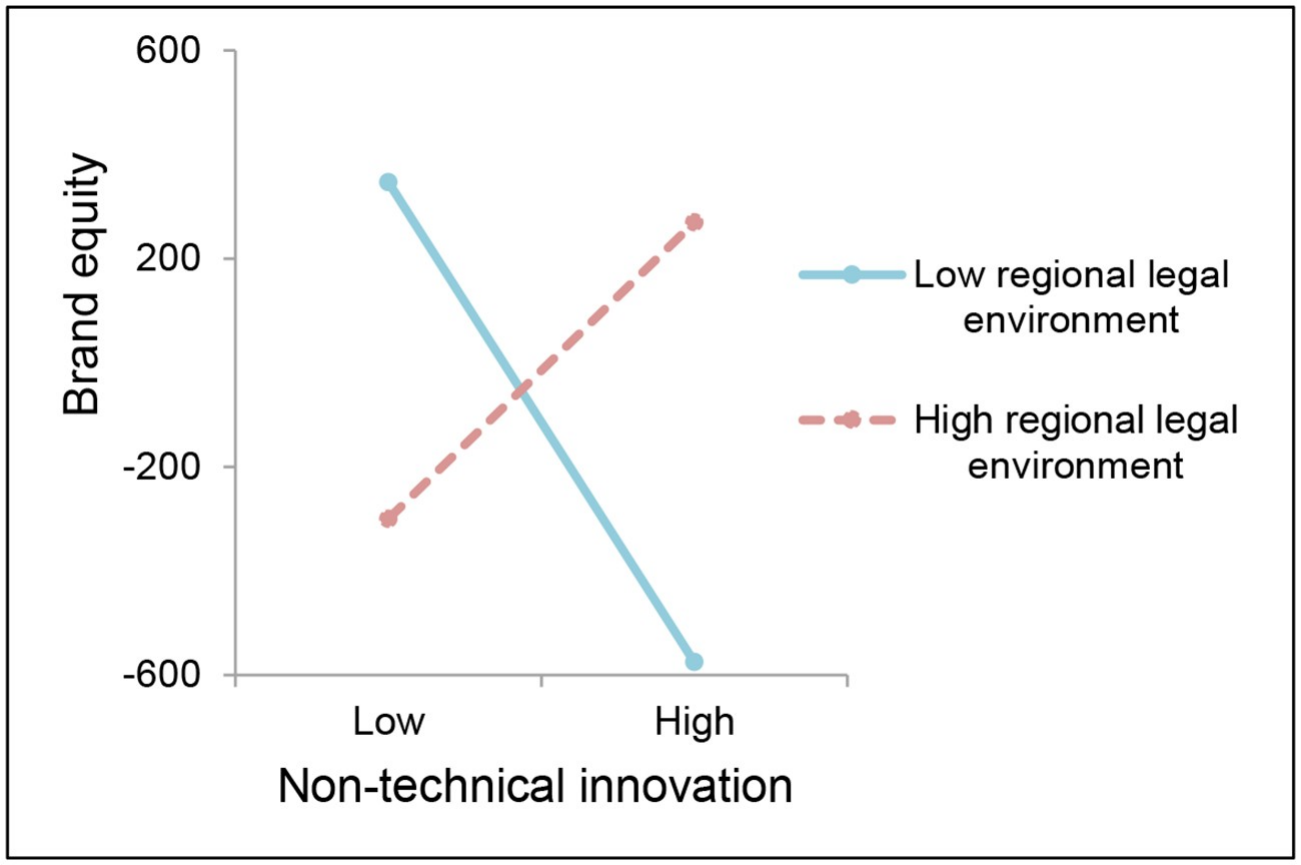
The moderating effects of H_4b_.

Next, we explore the extent of the specificity of the moderating effect of the regional legal environment. As predicted by H_4a_ and H_4b_, regarding whether technical innovation or non-technical innovation induces higher brand equity in regions with higher levels of legal environment (*β* = .002, *p* < .01; *β* = .002, *p* < .01, respectively), Fig 2 depicts that technical innovation has a stronger effect on brand equity when the regional legal environment is high, and the effect of non-technical innovation shows a similar pattern, as showed in Fig 3. It makes sense that a reliable legal system can help defeat the ‘hit-and-run’ imitation of innovation and provide protection of brand construction for firms.

## 5. Discussion

### 5.1 Theoretical implication

Based on the brand equity literature and institutional theory, this study offers empirical evidence to explore the effect of technical and non-technical innovation on the brand equity and the moderating effect of institutional factors, including product market development and regional legal environment, in the context of China—the emerging economy. Our analysis focuses on a set of panel data that may change the consumer perception on brands in terms of firm innovation strategies. Therefore, this study makes several important contributions to the innovation literature.

First, the extant literature provides an uncertain definition regarding the effect of innovation on firm performance (financial and non-financial performance). In particular, the study on the relation of innovation and brand equity is scarce. There is a good deal of research examining the innovation-performance relationship, but the conclusion whether it is positive or negative is changing within the dynamic context. Therefore, our study, which empirically considers the emerging economies context, is an important supplement because of the use of secondary multiple-source data. Additionally, most findings shed light on the technology-enabled innovations in the manufacturing industry, but research on people-enabled non-technical innovations is scant. In this paper, we observed the changes on brand that technical and non-technical innovations bring, and the empirical results that extend the brand equity literature highlight that the two types of innovation play pivotal roles in strengthening the brand equity.

Second, the innovation strategies (technical and non-technical) are contingent on the institutional environment, and institutional theory has been identified as a powerful theoretical lens in the strategy and management field [108]. This paper puts emphasis on the institution-specific characteristics of innovation by empirically investigating the moderating role of product market development and the regional legal environment. Prior scholars provide a number of contributions to the innovation literature in the context of developed countries characterized by complete institutional construction and law enforcement to prevent risks and uncertainty of markets. However, in emerging economies such as China, institutional voids severely inhibit a firm’s capability to appropriate values from new products. Additionally, there is much risk and uncertainty when innovative behaviors are introduced into practice, and uneven market development and imperfect law construction give rise to the unforeseeable contingencies such as unavoidable imitations and market failure. Hence, there is a gap researching how these drastic changes of institutions shape the relation of innovation and brand in the emerging economies such as China.

Of the institutional factors, in transitional China the product market development varies tremendously across the country. This complex mix of legal, administrative and marketing mechanisms may jeopardize the development of free market mechanisms [109], but in the innovation literature the dysfunctional competition effect is in research paucity. As we evidenced above, product market development strengthens the relationship between technical innovation and brand equity but limits the effect between non-technical innovation and brand equity. When product market development is high, the market competition intensifies, and non-technical innovation may not transform to the competitive advantages in the short run so that brand equity may not increase significantly.

Another factor regional legal environment is the crucial determinant which means a lot for market support and protection of innovation practices. Obviously, the transaction cost would increase when the regional legal system is incomplete. In addition, innovation under the law unenforceability is especially sensitive to copyright infringement of a weak legitimate construction. The findings in this study show that regional legal environment strengthens the performance effect of technical and non-technical innovation and brand equity.

### 5.2 Managerial implication

The findings offer important implications for innovative strategies for managers, especially in China. As the second largest economy in the world, the fast-growing Chinese market brings both opportunities and challenges to many multinational enterprises [36]. To capture the first-mover advantages in the drastic changing China, managers should realize the important role of innovation, which not only can deal rationally with institutional pressure but also increase the brand equity. In particular, the customers’ concern regarding a product has changed. They not only focus on whether the technical changes can meet their needs but also the people-enabled new interactions, so unnoticed non-technical innovation especially in the service sector may play a role in enhancing customer satisfaction because of its human-interaction nature. In addition to the financial values, such as sales and profit, it is possible for a firm to occupy the customer mindset through adequate innovative strategies. Because the resources and directions required to achieve the two innovative actions differ, a firm manager should determine how to assign strategies to capture and reinforce customer perception to optimize the brand equity.

Additionally, the decision has to weigh in the institutional environment. It is necessary to consider the impact of the external environment on firm behaviors. Managers must pay attention to the dynamic relationship between the market, competitors and consumers consistent with the dynamic institution-specific characteristics. A consideration of external institutional and market conditions can help managers resolve this dilemma [74]. Product market development will have stronger beneficial effects on the relation of technical innovation and brand equity, however it plays a relatively unimportant role in lifting the relation of non-technical innovation and brand equity. Therefore, managers should consider more technical innovations to optimize the interactions between employees and customers.

Moreover, firms should note the efficiency of the legal system when introducing innovations. In emerging economies, such as China still hosts an underdeveloped legal construction, thus firms often encounter difficulties in safeguarding their rights. For example, if legal construction cannot provide sufficient protection on firms’ intellectual property, such innovations may not transform to the superior outcomes and customer perception, which lead to the higher brand equity. Thus, managers may consider shifting the R&D center to the districts which the legal environment is better.

### 5.3 Limitations and further research

This study is subject to several limitations that demand future research attention. First, our sample is limited to firms in China. Countries vary in the level of different regional economic development and the multiple institutional characteristics that appear, and emerging economies vary markedly in their stages of economic and institutional development, so we suggest caution before generalizing our findings to other nations. For example, the marketization level in China is lower than that in most emerging economies [72]. A more comprehensive study, with samples from multiple emerging economies, could generate new findings about the contingent role of institutions.

Second, emerging economies are often characterized by industrial factors such as industry development speed or industry concentration. In this paper, we observed institutional factors as the moderating role of the relation between innovation and brand equity, and further research may extend the literature by examining more industry-specific variables.

Third, this study only examines the impact of technical and non-technical innovation on brand equity. Further research might examine the impact magnitude across the two innovations, and then investigate the how institutional factors shape the firms’ innovative strategies.

## Supporting information

S1 Table The data of all variables. (DOCX)

## References

[1] Melewar TC, Nguyen B. Five areas to advance branding theory and practice. Journal of Brand Management. 2014; 21:758–769.

[2] Sriram S, Balachander S, Kalwani MU. Monitoring the dynamics of brand equity using store-level data. Social Science Electronic Publishing. 2007; 71:61–78.

[3] Lodish LM, Mela CF. If brands are built over years, why are they managed over quarters? Harvard Business Review. 2007; 85:104–112. 17642129

[4] Aaker DA. Managing brand equity. The Free Press, New York 1991.

[5] Aaker DA. Measuring brand equity across products and markets. California Management Review. 1996; 38:102–120.

[6] Keller KL. Conceptualizing, measuring, and managing customer-based brand equity. Journal of Marketing. 1993; 57:1–22.

[7] Barone MJ, Jewell RD. The innovator’s license: a latitude to deviate from category norms. Journal of Marketing. 2013; 77:120–134.

[8] Sheng S, Zhou KZ, Li JJ. The effects of business and political ties on firm performance: evidence from China. Journal of Marketing. 2011; 75:1–15.

[9] He JX. The differences of brand equity between local companies and foreign companies: a positive study based on CBRQ scale. China Industrial Economy. 2006; 8:109–116.

[10] Fan XC. Analysis of brand equity and its measurement. Nankai Business Review. 2000; 1:9–15.

[11] Nguyen B, Yu X, Melewar TC, Gupta S. Critical brand innovation factors (CBIF): Understanding innovation and market performance in the Chinese high-tech service industry. Journal of Business Research. 2016; 69:2471–2479.

[12] Rennings K. Redefining innovation—eco-innovation research and the contribution from ecological economics. Ecological Economics. 2000; 32:319–332.

[13] Barone MJ, Jewell RD. The innovator’s license: a latitude to deviate from category norms. Journal of Marketing. 2013; 77:120–134.

[14] Walsh V. Design, innovation and the boundaries of the firms. Research Policy. 1996; 25: 509–29.

[15] Pakes A, Schankerman M. “An exploration into the determinants of research intensity” in R&D, Patents, and Productivity, Griliches Zvi (Ed.), University of Chicago Press, Chicago 1984; 209–232.

[16] Mank DA, Nystrom H E. Decreasing returns to shareholders from R&D spending in the computer industry. Engineering Management Journal. 2001, 13:3–8.

[17] Wang H, Kimble C. Innovation and leapfrogging in the Chinese automobile industry: examples from Geely, BYD, and Shifeng. Global Business & Organizational Excellence. 2013; 32:6–17.

[18] Zhang H, Ko E, Lee E. Moderating effects of nationality and product category on the relationship between innovation and customer equity in Korea and China. Journal of Product Innovation Management. 2013; 30:110–122.

[19] Chu S, Keh HT. Brand value creation: analysis of the interbrand-business week brand value rankings. Marketing Letters. 2006; 17:323–331.

[20] Damanpour F. Organizational innovation: A meta-analysis of effects of determinants and moderators. Academy of Management Journal. 1991; 34:555–590.

[21] Christensen C. The innovator’s dilemma. Harvard Business School Press, 1997.

[22] Narin Francis, Elliot, Perry Ross. Patents as indicators of corporate technological strength. Research Policy. 1987; 16:143–155.

[23] OECD: Meeting Global Challenges through Better Governance: International Co-operation in Science, Technology and Innovation. OECD: Paris 2012.

[24] Bessler W, Bittelmeyer C. Patents and the performance of technology firms: evidence from initial public offerings in Germany. Financial Markets and Portfolio Management. 2008; 22:323–356.

[25] Huang YC, Yang ML. Reverse logistics innovation, institutional pressures and performance. Management Research Review. 2014; 37:615–641.

[26] Jakhar SK. Stakeholder engagement and environmental practice adoption: The mediating role of process management practices. Sustainable Development. 2017; 25:92–110.

[27] Dyer JH. Effective interfirm collaboration: how firms minimize transaction costs and maximize transaction value. Strategic Management Journal. 1997; 18:535–556.

[28] Williamson OE. The Economic Institutions of Capitalism. Free Press, New York 1985.

[29] Luo Y. Are joint venture partners more opportunistic in a more volatile environment? Strategic Management Journal. 2007; 28:39–60.

[30] Peng MW. Institutional transitions and strategic choices. Academy of Management Review. 2003; 28:275–296.

[31] Farquhar PH. Managing brand equity. Journal of Advertising Research. 1990; 30:7–12.

[32] Stahl F, Heitmann M, Neslin SA. The Impact of Brand Equity on Customer Acquisition, Retention, and Profit Margin. Journal of Marketing. 2012; 76:44–63.

[33] Mizik N. Assessing the total financial performance impact of brand equity with limited time-series data. Journal of Marketing Research. 2014; 51:691–706.

[34] Simon CJ, Sullivan MW. The measurement and determinants of brand equity: a financial approach. Marketing Science. 1993; 12:28–52.

[35] Mullen M, Mainz A. Brand and balance sheet: Putting a price on protected products. Acquisition Monthly. 1989; 24:26–27.

[36] Leone RP, Rao VR, Luo AM, Mcalister L, Srivastava R. Linking brand equity to customer equity. Journal of Service Research. 2015; 9:125–138.

[37] Keller KL. Strategic brand management: building, measuring and managing brand equity, 2nd Edition, Upper Saddle River, NJ: Prentice Hall 2003.

[38] Damanpour F. Evan WM. Organizational innovation and performance: the problem of “organizational lag”. Administrative Science Quarterly. 1984; 29:392–409.

[39] Armbruster H, Bikfalvi A, Kinkel S, Lay G. Organizational innovation: the challenge of measuring non-technical innovation in large-scale surveys. Technovation. 2008; 28:644–657.

[40] Cummings TG, Srivastva S. Management of Work: A Sociotechnical System Approach. Kent, OH: Kent State University Press 1977.

[41] Trist E, Murray H. The Social Engagement of Social Science: A avistock Anthology. Philadelphia, PA: University of Pennsylvania Press 1993.

[42] Damanpour F, Walker RM, Avellaneda CN. Combinative effects of innovation types and organizational performance: a longitudinal study of service organizations. Journal of Management Studies. 2009; 46:650–675.

[43] Jiménez-Jiménez D, Sanz-Valle R. Innovation, organizational learning, and performance. Journal of Business Research. 2011; 64:408–417.

[44] Mothe C, Nguyenthi TU. Non-technological and technological innovations: do services differ from manufacturing? An empirical analysis of Luxembourg firms. International Journal of Technology Management. 2010; 57:397.

[45] Sirilli G, Evangelista R. Technological innovation in services and manufacturing: results from Italian surveys. Research Policy. 1998; 27:881–99.

[46] Dotzel T, Shankar V, Berry LL. Service innovativeness and firm value. Journal of Marketing Research. 2013; 50:259–276.

[47] Ngo LV, O’Cass A. Innovation and business success: the mediating role of customer participation. Journal of Business Research. 2013; 66:1134–1142.

[48] Démurger S. Infrastructure development and economic growth: an explanation for regional disparities in china? Journal of Comparative Economics. 2001; 29:95–117.

[49] Pettigrew AM. Longitudinal field research on change: theory and practice. Organization Science. 1990; 1:267–292.

[50] North DC. Institutions, institutional change and economic performance. Cambridge: Cambridge University Press 1990.

[51] Hoskisson RE, Eden L, Lau CM., Wright M. Strategy in emerging economies. Academy of Management Journal. 2000; 43:249–267.

[52] Scherer F. New perspectives on economic growth and technological innovation. Brookings Institution Press: Washington, D.C. 1999.

[53] Walder AG. Property-rights and stratification in socialist redistributive economies. American Sociological Review. 1992; 57.

[54] Dacin MT, Oliver C and Roy J. The legitimacy of strategic alliances: An institutional perspective. Strategic Management Journal. 2007; 28:169–87.

[55] Suchman MC. Managing legitimacy: strategic and institutional approaches. Academy of Management Review. 1995; 20:571–610.

[56] Zimmerman MA, Zeitz GJ. Beyond survival: achieving new venture growth by building legitimacy. Academy of Management Review. 2002; 27:414–431.

[57] Jennings PD, Zandbergen PA. Ecologically sustainable organizations: an institutional approach. Academy of Management Review. 1995; 20:1015–1052.

[58] Chan CM, Makino S, Isobe T. Does subnational region matter? Foreign affiliate performance in the United States and China. Strategic Management Journal. 2010; 31:1226–1243.

[59] Young A. The razor’s edge: distortions and incremental reform in the People’s Republic of China. Quarterly Journal of Economics. 2000; 115:1091–35.

[60] Lockett M. China’s special economic zones: the cultural and managerial challenges. Journal of General Management. 1987; 12:21–32.

[61] Damanpour F. The adoption of technological, administrative, and ancillary innovations: impact of organizational factors. Journal of Management. 1987; 13:675–688.

[62] Pavitt K. The objectives of technology policy. Science & Public Policy. 1987; 14:182–188.

[63] Diaconu M. Technological innovation: concept, process, typology and implications in the economy. Theoretical & Applied Economics. 2011; 18:127–144.

[64] Ali M, Park K. The mediating role of an innovative culture in the relationship between absorptive capacity and technical and non-technical innovation. Journal of Business Research. 2016; 69:1669–1675.

[65] Schumann JH, Wünderlich NV, Wangenheim F. Technology mediation in service delivery: a new typology and an agenda for managers and academics. Technovation. 2012; 32:133–143.

[66] Paluch S, Blut M. Service separation and customer satisfaction: assessing the service separation/customer integration paradox, Journal of Service Research. 2013; 16:415–427.

[67] Blundell R, Griffith R, Reenen JV. Market share, market value and innovation in a panel of british manufacturing firms. Review of Economic Studies. 1999; 66:529–54.

[68] Park CS, Srinivasan V. A survey-based method for measuring and understanding brand equity and its extendibility. Journal of Marketing Research. 1994; 31:271–288.

[69] Bower JL, Christensen CM. Disruptive technologies: catching the wave. Harvard Business Review. 1995; 38:60–77.

[70] Beverland MB, Napoli J and Farrelly F. Can all brands innovate in the same way? A typology of brand position and innovation effort, Journal of Product Innovation Management. 2010; 27:33–48.

[71] Avlonitis GJ, Papastathopoulou PG, Gounaris SP. An empirically-based typology of product innovativeness for new financial services: success and failure scenarios. Journal of Product Innovation Management. 2001; 18:324–342.

[72] Ahlstrom D, Bruton GD. Rapid institutional shifts and the co-evolution of entrepreneurial firms in transition economies. Entrepreneurship Theory & Practice. 2010; 34:531–554.

[73] Clayton T. Service innovation: Aiming to win. Imperial College Press, London 2003.

[74] Storey C, Kelly D. Measuring the performance of new service development activities. Service Industries Journal. 2001; 21:71–90.

[75] Hagedoorn J, Roijakkers N, Kranenburg V. Interfirm R&D networks: The importance of strategic network capabilities for high-tech partnership formation. British Journal of Management. 2006; 17:39–53.

[76] Camisón C, Villar-López A. Non-technical innovation: organizational memory and learning capabilities as antecedent factors with effects on sustained competitive advantage. Industrial Marketing Management. 2011; 40:1294–1304.

[77] Bendapudi N, Leone RP. Psychological implications of customer participate on in co-production. Journal of Marketing. 2003; 67:14–28.

[78] North DC. Explaining the swollen middle: why most transactions are a mix of “market” and “hierarchy”. Organization Science. 1993; 4:529–547.

[79] Waarden BFV. Institutions and innovation: the legal environment of innovating firms. Organization Studies. 2001; 22:765–795.

[80] Haeussler C, Patzelt H, Zahra SA. Strategic alliances and product development in high technology new firms: The moderating effect of technological capabilities. Journal of Business Venturing. 2012; 27:217–233.

[81] Jaworski BJ, Kohli AK. Market orientation: antecedents and consequences. Journal of Marketing. 1993; 57:53–70.

[82] Stock GN, Greis NP, Fischer WA. Firm size and dynamic technological innovation. Technovation. 2002; 22:537–549.

[83] Oliver C. Sustainable competitive advantage: combining institutional and resource-based views. Strategic Management Journal. 1997; 8:679–713.

[84] Brown SL, Eisenhardt KM. Product development: Past research, present findings, and future directions. Academy of Management Review. 1995; 20:343–378.

[85] Lundvall BÅ. National innovation systems—analytical concept and development tool. Industry & Innovation. 2007; 14:95–119.

[86] Ahuja G, Lampert CM, Tandon V. Moving beyond Schumpeter: management research on the determinants of technological innovation. Academy of Management Annals. 2008; 2:1–98.

[87] Nieto M, González-Álvarez N. Product innovation: testing the relative influence of industry, institutional context and firm factors. Technology Analysis & Strategic Management. 2014, 26:1023–1036.

[88] Cohen WM, Nelson RR, Walsh JP. Protecting their intellectual assets: appropriability conditions and why U.S. Manufacturing Firms Patent (or not). Cambridge, MA: NBER 2000.

[89] Haunschild PR., Miner AS. Modes of interorganizational imitation: the effects of outcome salience and uncertainty. Administrative Science Quarterly. 1997; 42:472–500.

[90] Berrone P, Fosfuri A, Gelabert L, Gomez‐Mejia LR. Necessity as the mother of “green” inventions: institutional pressures and environmental innovations. Strategic Management Journal. 2013; 34:891–909.

[91] Hargadon AB, Douglas Y. When innovations meet institutions: Edison and the design of the electric light. Administrative Science Quarterly. 2001; 46:476–501.

[92] Lieberman MB, Montgomery DB. First-mover (dis) advantages: Retrospective and Link with the Resource-based View. Strategic Management Journal. 1998; 19:1111–1125.

[93] Chang SJ, Wu B. Institutional barriers and industry dynamics. Strategic Management Journal. 2014; 35:1103–1123.

[94] Jia N. Are collective political actions and private political actions substitutes or complements? Empirical evidence from China’s private sector. Strategic Management Journal. 2014; 35:292–315.

[95] Berelson B. Content analysis in communication research. American Political Science Association. 1952; 46:869.

[96] Kerlinger FN. Foundations of behavioural research (3rd ed). Orlando, FL: Hold, Rinehart and Winston 1986.

[97] Belkaoui A, Belkaoui JM. A comparative analysis of the roles portrayed by women in print advertisements: 1958, 1970, 1972. Journal of Marketing Research. 1976; 13:168–172.

[98] Humphreys A, Latour KA. Framing the game: assessing the impact of cultural representations on consumer perceptions of legitimacy. Journal of Consumer Research. 2013; 40:773–795.

[99] Philippe D, Durand R. The impact of norm‐conforming behaviors on firm reputation. Strategic Management Journal. 2011; 32:969–993.

[100] Riffe D, Lacy S, Fico F. Analyzing media messages: using quantitative content analysis in research. Routledge/Taylor & Francis Group, 2014.

[101] Scott WA. Reliability of content analysis: the case of nominal scale coding. Public Opinion Quarterly. 1955; 19:321–325.

[102] Cohen J. A coefficient of agreement for nominal scales. Educational & Psychological Measurement. 1960; 20:37–46.

[103] Li D, Cao C, Zheng M, Huang M, Ren S, Chen X. The impact of legitimacy pressure and corporate profitability on green innovation: Evidence from China top 100. Journal of Cleaner Production. 2017; 141:41–49.

[104] Semykina A, Wooldridge JM. Estimating panel data models in the presence of endogeneity and selection. J. Econom. 2010; 157: 375–380.

[105] Nickell S. Biases in Dynamic Models with Fixed Effects. Econometrica. 1981; 49:1417–1426.

[106] Chen Y. Factors influencing renewable energy consumption in China: An empirical analysis based on provincial panel data. Journal of Cleaner Production. 2017; 174:605–615.

[107] Baum C F, Schaffer M E, Stillman S. Instrumental variables and GMM: Estimation and testing. Stata Journal. 2003; 3:1–31.

[108] Barbosa N, Faria AP. Innovation across Europe: How important are institutional differences? Research Policy. 2011; 40:1157–1169.

[109] Tan J, Li S, Xia J. When iron fist, visible hand, and invisible hand meet: firm-level effects of varying institutional environments in china. Journal of Business Research. 2007; 60:786–794.

