# Supplementary material for: The effects of tech and non-tech innovation on brand equity in China: The role of institutional environments
Source: PLoS One. 2019 May 8;14(5):e0215634. doi: 10.1371/journal.pone.0215634 (PMC6505746; doi:10.1371/journal.pone.0215634)
Supplement: S1 Table — (DOCX) [file pone.0215634.s001.docx]

# Supporting information

**S1 Table. The data of all variables**

Note: please double click the following table to see more details.
